# Cobalt-Induced Toxicity and Spasticity Secondary to Hip Arthroplasty: Case Report and Review of the Literature

**DOI:** 10.7759/cureus.12368

**Published:** 2020-12-29

**Authors:** Vishal Venkatraman, Megan K Wong, Chidyaonga Shalita, Beth Parente, Shivanand P Lad

**Affiliations:** 1 Department of Neurosurgery, Duke University Medical Center, Durham, USA

**Keywords:** cobalt toxicity, spasticity, hip arthroplasty, metallosis

## Abstract

Cobalt is known to produce a variety of symptoms in patients who accumulate a toxic amount in their blood. Cobalt poisoning can arise from metal implants due to wear and tear on the metal implant surfaces, but implant deterioration has not yet been reported to cause muscle spasticity. A 45-year-old male patient with a medical history of multiple sclerosis (MS) and bilateral hip arthroplasty presented with spasticity that persisted despite administration of anti-spasmodic medication and intrathecal baclofen. Concerns of high cobalt levels, confirmed via blood testing, led to revisions of both of his hip prosthesis, which alleviated his muscle spasms. To our knowledge, this is the first reported case of muscle spasticity associated with increased blood cobalt levels. Reduction in the patient’s spasticity was associated with prosthesis revision and subsequent reduction in blood cobalt, suggesting that cobalt was involved in the pathogenesis or at minimum worsening of his spasticity given his concurrent MS. Review of the literature suggests that increased levels of cobalt can interfere with metabolism in neurons and damage muscle fibers, providing possible pathological mechanisms for the patient’s spasticity.

## Introduction

Cobalt is an essential trace element that has important roles in the production and regulation of red blood cells, platelets, and DNA as well as fatty acid synthesis and energy production. Normal daily dietary intake ranges from 5 to 50 μg, with normal plasma concentrations of <0.2 μg/L. Excessive cobalt can produce systemic toxicity affecting multiple organ systems. Adverse effects related to cobalt toxicity can occur at levels of 7-10 μg/L or more. Previously, cobalt toxicity has been seen in the form of pulmonary disease in those who inhaled cobalt dust while drilling or polishing. More recently, systemic cobalt toxicity has been associated with metal-on-metal (MoM) total hip arthroplasty or shaving of cobalt-chromium secondary to retained ceramic particles from a failed femoral head prosthesis, causing the release of cobalt and chromium metal ions into systemic circulation. Post-arthroplasty metallosis with systemic cobalt toxicity is a rare complication, but morbidity and mortality are high.

Severe spasticity has not been previously reported in patients with cobalt toxicity secondary to prosthetic implants. Systemic toxic effects from excessive levels of cobalt include peripheral neuropathy, sensorineural hearing loss, vision loss, cognitive decline, cardiomyopathy, hypothyroidism, weakness, fatigue and polycythemia. Although there is not yet a proven causal link, these reported symptoms are consistent with those of cobalt poisoning [1]. Neuromuscular symptoms are rare, but include reports of decreased muscle mass, tremor, and convulsions [2]. We present a complex case of patient with multiple sclerosis (MS) and severe spasticity, treated with neurosurgical interventions including intrathecal baclofen pump, who was later found to have systemic cobalt toxicity that improved with total hip arthroplasty revision surgeries. We propose an underlying mechanism of the spasticity due to complications from metallic hip prosthesis.

## Case presentation

A 45-year-old male presented to our institution for evaluation and treatment of severe generalized spasticity. His past medical history was significant for right hip arthroplasty with a cobalt-chrome implant in 2010, left hip arthroplasty with a cobalt-chrome implant in 2011, and relapsing-remitting multiple sclerosis, diagnosed in 2013 with scattered demyelinating lesions in his brainstem, brain and spine. At the time of presentation, the patient reported MS-related symptoms of muscle stiffness, balance difficulties, vision problems, altered sensations, and transient body-wide stabbing pains in his joints, worst at the wrists and left calf. He also reported episodic spasms of his full-body, worse in the legs, as well as difficulty breathing.

The patient was managed with antispasmodic medications (gabapentin 200mg x2/day; diazepam 5mg 4x/day; lorazepam 1mg 2-4x/day; oxcarbazepine 600mg 2x/day; tizanidine 4-8 mg/day) all of which provided minimal symptom relief. He also took an MS disease-modifying drug (natalizumab 300mg/15mL injection per month). The patient had attempted oral baclofen, but this was not tolerated. Given his lack of improvement with conservative treatment, the patient was considered an appropriate candidate for an intrathecal baclofen tunneled-catheter trial. 

An intrathecal trial catheter was placed under fluoroscopic guidance into the intrathecal space and was guided to the T6 vertebral body. At two-day post-procedure, the patient was returned to the operating room for a revision due to occlusions in catheter infusion flow. A small segment of the catheter extension was trimmed, and a larger gauge needle attachment was added, providing brisk CSF flow. The patient’s intrathecal baclofen rate was titrated to 150mcg/24hr over the next week, at which was found to be the level at which the patient neither experienced full-body spasms nor flaccid paralysis. The patient elected to move forward with the implantation of a programmable pump for baclofen infusion, which was uneventful, and he was discharged home the following day.

At five-month post-procedure, the patient reported a complete absence of his prior spasmodic episodes with his baclofen dose at 161 mcg/24hr. At this time, he was also taking gabapentin 600mg 3x/day; diazepam 5mg in the morning, 5mg mid-day, 15 mg in the evening, and 2.5 mg every six hours PRN; oxcarbazepine 300mg 2x/day; natalizumab 300mg/15mL injection per month; tizanidine 3 mg 2x/day. By seven months post-procedure, the patient began to experience spasms that worsened during the evening hours despite up-titration of his baclofen pump rate to 180 mcg/24hr. 

At 10-month post-procedure, the patient was hospitalized twice due to episodes of alternating flaccidity and rigidity. During the first episode he was intubated and there was a concern for baclofen withdrawal, but after interrogation, there were no abnormal flow issues and his symptoms gradually resolved. The pump settings were reduced and changed to a simple continuous mode which was tolerated, and he was discharged. At the time of discharge intrathecal baclofen rate was 176mcg/24hr.

During the second episode, the patient was admitted once again with similar symptoms of alternating flaccidity and rigidity. Due to the unknown etiology of his symptoms, the patient was taken to the operating room and underwent a revision of the baclofen pump and a catheter replacement. Intraoperative findings displayed coiling near the catheter attachment site that may have kinked and blocked baclofen flow. The patient was neurologically intact postoperatively and discharged home.

**Figure 1:**
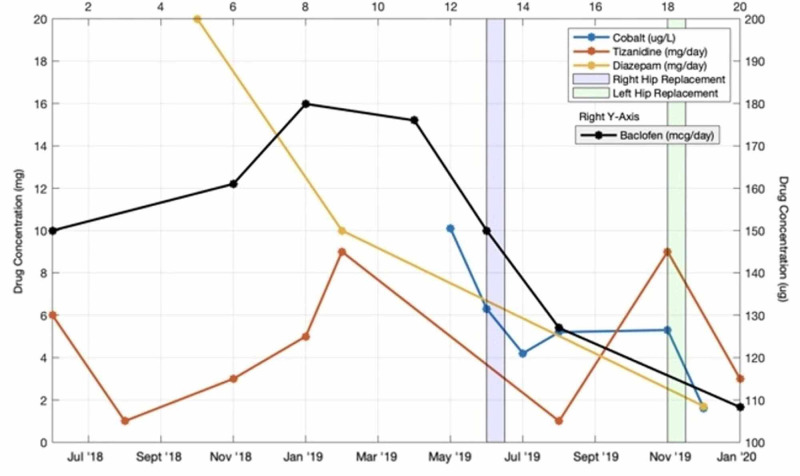
Changes in anti-spasmodic medications and blood cobalt over time.

Following the pump revision, the patient continued to experience spasms, now accompanied by worsening pain in his hips bilaterally. This raised the concern about metallosis and it was found that his blood cobalt level was 10.1ug/L and that the trunnion, or head-neck joint, of his right hip implant had worn down when viewed on X-ray imaging. He underwent an initial right hip revision to a ceramic implant and his cobalt levels decreased to 6.3ug/L. One-month post-surgery, the patient’s cobalt levels dropped further to 4.2ug/L and he reported a decrease in pain and improvement in his hip pain. However, the patient experienced a recurrence of spasticity and left hip pain over the next few months. After workup revealed elevated cobalt levels of 5.3ug/L and wear of the left trunnion, the patient underwent a left hip arthroplasty revision. Following this, the patient reported significant reductions in his spasticity and hip pain, and his blood cobalt level decreased rapidly to 1.6 ug/L. His symptoms of spasticity were now only significant for tightness across the shoulders at night that woke him up from sleep. His baclofen pump rate was reduced to 108mcg/24hr, his tizanidine was reduced to 3mg/day, and his diazepam was reduced to 5mg about once every three days, as needed. The changes in the patient’s medications and blood cobalt levels over the course of his treatment are shown in Figure 1.

## Discussion

Our patient presented with severe spasticity secondary to cobalt toxicity, which was thought to be secondary to his MS initially. To date, there have been various presentations of cobalt toxicity, but associations between cobalt toxicity and spasticity have not been previously described. Here, we present a unique patient whose elevated blood cobalt level correlated with considerable increase in spasticity, which improved after bilateral hip arthroplasty revision surgery.

The diagnosis of cobalt toxicity requires recognizing the potential of hip implant failure to cause worsening neurological symptoms, including worsening spasticity, in addition to other systemic symptoms. In particular, a detailed surgical history should be obtained in all patients with unexplained spasticity and cobalt toxicity should be ruled out in patients with cobalt implants. Elevated serum levels of cobalt will confirm the clinical suspicion. There is no proven treatment for cobalt toxicity aside from reducing blood cobalt levels by either revising or replacing the failed hip arthroplasty as soon as it is recognized.

Due to his MS, the patient suffered from spasticity, but there was also a noticeable correlation between the patient’s blood cobalt and worsening spasticity. After hip arthroplasty revision and his cobalt levels decreased, the patient was able to control his spasticity with lower doses of medications. The reduction in the patient’s spasticity pre-and-post hip revision can be quantified with the Ashworth Scale, a clinical tool commonly used to assess spasticity in patients with neurological conditions including MS [3]. Pre-revision, the patient’s Ashworth matched the criteria for a maximum score of 4 in multiple body joints, with affected limbs in rigid flexion and extension. Post-revision of both hips, his spasticity matched a score of 3, or a considerable increase in muscle tone, but now only in the shoulders, as opposed to throughout the body. There is not a clear consensus on the cobalt levels required to cause toxicity, but a review by Paustenbach et al. [4] mentions that current guidelines suggest a safety threshold of 7-10ug/L, and that levels over 10ug/L can indicate significant hip prosthesis wear.

The exact mechanisms of cobalt toxicity have not been identified, but various mechanisms have been proposed which may explain our patient’s presentation. The inhibition of the Krebs Cycle may play a role in the development of systemic muscular spasticity in the presence of cobalt toxicity [5]. Neurons have a high energy consumption due to their role in signal propagation in the nervous system. In turn, dysfunction at the mitochondrial level may result in a breakdown of organized electrical signal transmission along neurons resulting in the observed episodes of spasticity and flaccidity seen in our patient. 

In a review on neurological effects of cobalt, it was proposed that neural damage may be caused by cobalt-induced mitochondrial damage [6]. It has been shown that cobalt leads to the production of reactive oxygen species [6], which could lead to mitochondrial and DNA damage in neurons. This mechanism could also account for symptoms similar to the Krebs cycle interruption mentioned previously. 

Long-term low-level exposure of cobalt also can result in the displacement of calcium-binding in the peripheral nervous system and can lead to a wide range of neurological symptoms including paresthesia, hearing loss, and peripheral vision loss [5]. Calcium plays a pivotal role in the transmission of neurological signals via the transmission of action potentials at the axon terminal involving calcium-mediated neurotransmitter release. A disruption in this important signaling system can lead to the development of neurological disorders. In the context of our patient developing systemic muscular spasticity, it is possible that the accumulation of cobalt ions in his blood resulted in the disruption of neuronal signaling manifesting in intense episodes of spasticity paired with intermediate episodes of flaccidity. 

Another mechanism of the cobalt-induced spasticity seen in our patient could be direct damage to muscle fibers. It has been shown that high levels of Cobalt (CoCl2) cause cytotoxicity in muscle cells, leading to muscular atrophy and dysfunction [7]. Sustained cobalt exposure increases activation of necrotic factors and pro-inflammatory factors in a dose-dependent manner, causing myotubule atrophy that worsens with higher doses. Another study showed that CoCl2 can mimic hypoxic conditions and lead to the activation of autophagic pathways in muscle cells, causing cell atrophy through degradation of muscular proteins [8]. The release of cellular degermation factors might be responsible for aberrant firing of muscle cells or irregular reaction to nerve stimulation, leading to irregular contraction and spasms. 

Because of the risk of developing systemic metal ion toxicity, the Food and Drug Administration (FDA) currently recommends patients with symptoms suggestive of hip arthroplasty failure or signs of systemic symptoms to seek evaluation by an orthopedic surgeon for imaging of the hip implant and confirmation of serum cobalt levels [9]. Furthermore, the FDA recommends that asymptomatic patients with MoM hip prosthesis follow up regularly with their orthopedic surgeon [10]. Patients with a history of hip arthroplasty, who subsequently develop unexplained neurologic symptoms including severe spasticity, should be screened for metallosis and cobalt toxicity

## Conclusions

We presented a case of muscle spasticity in a patient with bilateral hip replacements and underlying multiple sclerosis. The patient presented with increased blood cobalt, leading to revisions of both hips, reduction in cobalt levels, and subsequent improvement in spasticity symptoms. Review of the literature suggests that cobalt toxicity could lead to spasticity by disrupting neuronal metabolism, interfering with neuronal signaling, and damaging muscle fibers. This case should prompt further inquiry into the safety of metal prostheses and additional screening for arthroplasty patients with a similar clinical presentation.
